# PPD in HBsAg vaccine formulation suppressed IFN-γ and IL-4 cytokine responses and induced long-lived humoral immune responses: Results from 220-day monitoring of specific IgG responses

**DOI:** 10.22038/IJBMS.2022.61941.13710

**Published:** 2022-11

**Authors:** Rayhaneh Mirzaee, Fahimeh Nemati, Mina Mirzaee, Bahareh Golkaran, Akbar Khorasani, Niloufar Rashedi, Fatemeh Asgarhalvaei, Mohammad Ali Savoji, Mehdi Mahdavi

**Affiliations:** 1 Department of Biotechnology, Faculty of Advanced Sciences & Technology, Tehran Medical Sciences, Islamic Azad University, Tehran, Iran; 2 Department of Microbiology, Faculty of Advanced Sciences & Technology, Tehran Medical Sciences, Islamic Azad University, Tehran, Iran; 3 Department of FMD vaccine, Razi Vaccine and Serum Research Institute, Agricultural Research, Education and Extension Organization (AREEO), Karaj, Iran; 4 Department of Biology, Faculty of basic science, Shahrekord Branch, Islamic Azad University, Shahrekord, Iran; 5 Advanced Therapy Medicinal Product (ATMP) Department, Breast Cancer Research Center, Motamed Cancer Institute, Academic Center for Education, Culture and Research (ACECR), Tehran, Iran; 6 Recombinant Vaccine Research Center, Tehran University of Medical Sciences, Tehran, Iran; 7 Immunotherapy Group, The Institute of Pharmaceutical Sciences (TIPS), Tehran University of Medical Sciences, Tehran, Iran; 8 Department of Immunology, Pasteur Institute of Iran, Tehran, Iran

**Keywords:** Adjuvant, HBs antigen, Long-lived IgG response, MF59, PPD

## Abstract

**Objective(s):**

Here, immune responses and long-lived IgG responses of HBsAg-Alum, HBsAg-MF59, as well as HBsAg-MF59 were compared when formulated with PPD.

**Materials and Methods:**

BALB/c mice were vaccinated subcutaneously three times with a two-week -interval. Then, specific IgG, long-lived IgG responses up to 220 days, and IgG1/IgG2a isotypes, and IFN-γ and IL-4 on spleen cell culture supernatant were assessed using ELISA.

**Results:**

IFN-γ cytokine response between MF59- and Alum-adjuvanted vaccines did not show a significant difference. HBsAg-Alum revealed an increase in IL-4 cytokine versus HBsAg-MF59 at borderline (*P*=0.0553). In addition, HBsAg-MF59+PPD 10 µg showed a significant decrease in IL-4 and IFN-γ cytokines versus HBsAg-MF59. Furthermore, HBsAg-MF59+PPD10 µg showed a significant increase in the IL-2/IL-4 ratio versus HBsAg-MF59 (*P*=0.0339). Specific IgG antibody showed a significant increase in HBsAg-MF59, as compared with HBsAg-Alum. Furthermore, HBsAg-MF59 plus PPD showed a significant increase in IgG responses versus HBsAg-MF59 and HBsAg-Alum groups. Long-lived IgG responses showed a significant increase in HBsAgMF59 versus HBsAg-Alum group and PPD in the HBsAg-MF59 vaccine formulation, resulting in a significant increase in IgG responses versus HBsAg-MF59 group. In addition, HBsAg-MF59 plus PPD suppressed IgG1 response versus HBsAg-Alum. However, HBsAg-MF59 showed a significant increase in IgG2α versus the HBsAg-Alum group (*P*=0.0190). Immunization with HBsAg-MF59+PPD (10 µg) showed a significant increase versus the HBsAg-MF59 group (*P*=0.0040). IgG2a/IgG1 ratio in HBsAg-MF59+PPD1µg and HBsAg-MF59+PPD10 µg groups showed a significant increase versus HBsAg-MF59 groups (*P*<0.0345).

**Conclusion:**

PPD leads to a more potent long-lived IgG responses in the HBsAg vaccine, highlighting its potential as a component of a complex adjuvant.

## Introduction

The *Hepadnaviridae* family including the hepatitis B virus is known for double-strand genomic DNA which is 42 nm in diameter and has two outer capsids and replicates in the liver and causes liver failure. The external capsid of the virus consists of lipids and surface antigens (HBsAg). Viral hepatitis is a systemic disorder that primarily affects the liver and causes inflammation of the liver, fever, nausea, vomiting, and jaundice (1-3).

 About 10000 people in Iran are infected with this virus each year, hence hepatitis B is one of the main liver diseases in this country (4). Vaccination is one of the most successful medical methods in the protection against infectious diseases (5-7). There is no doubt that the vaccine strategy is much more cost-effective than the treatment strategy. In addition, the cost of vaccination for each disease is less than the cost of treatment with effective drugs (5, 8). Today, recombinant protein-based vaccine is applied to prevent hepatitis B infection formulated in alum hydroxide adjuvant. The appropriate response to HBsAg is strongly related to the collaboration of T-cells and also, the interaction between T helper cells and B lymphocytes (9, 10). It has been proven in mouse models that many MHC-II haplotypes are incompatible with HBsAg presentation. In other words, the HBsAg has no proper structure for these haplotypes, and MHCs will not be able to bind to the T-cell antigenic index and will not present it (11). Adjuvants are substances that increase the immunogenicity of immunogens. Adjuvants were introduced in the early 1920s and at present, their numbers and functions have been expanded greatly. Alum is the most widely-used adjuvant capable of producing powerful humoral immune responses; however, alum is not capable of stimulating cellular immune responses and is associated with complications such as fever and chills and related nervous system diseases (12). Moreover, the effectiveness of the HBsAg vaccine was demonstrated to be unacceptable in some recipients (13). Therefore, it seems that changing the hepatitis B vaccine formulation may increase the efficiency of the vaccine in these individuals. MF59 adjuvant is an oil-in-water adjuvant, which is structured from squalene and surfactant which has tween 80 and span 85. This adjuvant was first used in 1997 in the seasonal influenza vaccine and significantly increased the immunogenicity in adolescents and aged individuals. The adjuvant exerts its activity through the increase and recruitment of immune cells to the injection site in order to trigger immune responses (14). 

Studies showed that PPD as the extract of *Mycobacterium tuberculosis* is able to reinforce DC function and trigger inflammatory responses (15). This property is important in the recruitment of immune cells in the injection site and increases immunogenicity against antigens. In this regard, we hypothesized that using MF59 adjuvant and PPD in HBsAg formulation, may affect the humoral and cellular immune responses and gives a higher response in comparison with Alum-based vaccines and even MF59-based vaccines. In addition, long-lived IgG responses were assessed up to 220 days post-final immunization.

## Materials and Methods

***Vaccine formulation***

The Alum-based HBsAg vaccine and purified recombinant HBsAg protein were provided by the Production Complex of Pasteur Institute of Iran, Karaj, Iran. Regarding HBsAg formulation in MF59 adjuvant, the recombinant HBsAg protein was added to MF59 adjuvant at the ratio of 50:50 and mixed for 30 min by vortexing in a cleanroom. After preparation of HBsAg-MF59, PPD (Razi Vaccine and Serum Research Institute, Karaj, Iran) at 1 and 10 µg doses was added to the HBsAg-MF59 formulation and further mixed for 10 min. In the final product of the vaccine, each 100 µl of the vaccine contained 5 µg of HBsAg vaccine either with 1 and /or 10 µg of PPD used for immunization.

***Experimental animals***

Ninety inbred female Balb/c mice (6–8 weeks old) were purchased from Pasteur Institute of Iran (Karaj). One week before the experiment mice were housed at 20–22 °C with appropriate ventilation with unlimited access to food and water and a standard light/dark cycle (12 hr/12 hr) in the animal room of Pasteur Institute of Iran. All experiments were performed according to the Animal Care and Use Protocol of the Ethics Committee of the Islamic Republic of Iran (code IR.IAU.PS.REC.1397.215).

***Mouse grouping and immunization***

Nine experimental groups of mice containing 10 mice in each group were used. They were immunized subcutaneously on days 0, 14, and 28 with 100 μl of HBsAg (5 μg) formulated in MF59 adjuvant, HBsAg-MF59+PPD 1 and or/10 µg, commercial HBsAg-Alum vaccine with the same condition. Some experimental mice were injected with MF59, MF59+PPD 1 and or/10 µg, Alum, and PBS with the same protocol as the control groups.

***IFN-γ and IL-4 cytokines responses***

Sterile cold PBS containing 2% FBS and pen/strep was used to suspend the removed spleen cells from mice two weeks after the final immunization. Red blood cells were lysed using lysis buffer and after centrifugation remaining cells were resuspended to be adjusted to 3×10^6^ cells/ml in RPMI-1640 (Gibco, Germany) supplemented with 5 % FBS, 4 mM L-glutamine, 1 mM sodium pyruvate, 100 µg/ml streptomycin and 100 IU/ml penicillin. 3×10^6^ cells were suspended in 1 ml of and cultured in each well of a 24-well plate and treated with 5 μg/ml of HBsAg for 60 hr at 37 °C and 5% CO_2_. The supernatant of each group of cells was then collected to be used for measurement of IFN-γ and IL-4 cytokines assay using ELISA kit (Mabtech, Sweden) and quality control using standard group and reported as pg/ml according to the manufacturer’s instructions. In addition, the IL-2/IL-4 cytokine ratio was determined using the raw data of each one.

***Specific total IgG responses***

About two weeks after the third immunization, the sera were collected from the mice and specific total IgG antibodies were evaluated using an optimized indirect ELISA. In addition, long-lived antibody responses were monitored on days 90, 150, and 220 after the final shot. Briefly, 100 µl of HBsAg at a concentration of 5 µg/ml in PBS was coated in 96-well ELISA Maxisorp plates (Greiner, Germany) and incubated at 4 °C overnight. Using washing buffer (0.05% Tween 20 in PBS) wells were washed 3 times followed by blocking buffer (2% skimmed milk and 0.05% Tween 20 in PBS) for 1 hr at 37 °C. Then, dilution buffer (1% PBS-BSA containing 0.05% Tween 20) was prepared to perform sera serial dilutions in order of 1/25 to 1/838860800. One hundred microliters of each dilution were added to each well and incubated at 37 °C for 2 hr. Subsequently, 100 µl of 1/8000 dilution of anti-mouse conjugated to HRP (Sigma, USA) was added to the wells following washing five times with washing buffer and incubated for further 2 hr at 37 °C. After being washed five times with washing buffer, 100 µl of TMB substrate (Razirad, Iran) was added followed by incubation for another 30 min in the darkness. The reaction was stopped using 100 µl of 2N H_2_SO_4_ and the absorption wavelength was measured at 450/630 nm with an ELISA plate reader (AWARENESS technology, USA). In addition, two weeks after the third immunization, collected sera were used for measurement of specific IgG1 and IgG2a antibodies and were assessed using goat anti-mouse IgG1 and IgG2a secondary antibodies (Sigma, USA) in accordance with the manufacturer’s instruction. In addition, the IgG2α/IgG1 ratio, for each one of the mice was evaluated and reported.

***Statistical analysis***

All experiments were performed in duplicate and /or triplicate. The mean of each duplicate and /or triplicate was calculated and presented as Mean±S.D. Data analysis was performed using Graph Pad prism V6.01 software. The one-way ANOVA method and Tukey’s multiple comparisons test as a *post hoc* test were used to determine the statistical significance among the mice different groups. For IgG1 and IgG2a isotype antibodies, the Mann-Whitney U test was used. *P*-values less than 0.05 were assumed significant.

## Results

***Results of IFN-γ cytokine***

**Figure 1:**
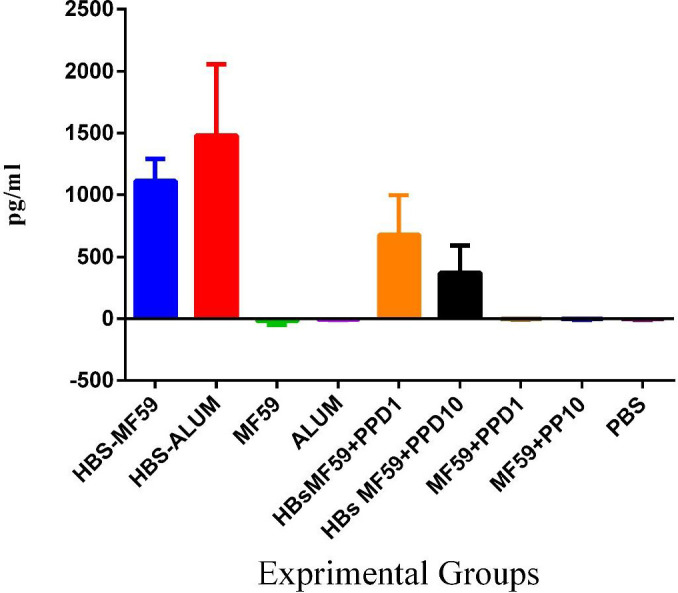
IFN-γ cytokine response in the experimental groups. Mice immunized with HBsAg-Alum showed no significant difference compared with HBsAg-MF59 (*P*=0.2300). Mice immunized with HBsAg-MF59 vaccine plus PPD at the doses of 1 and 10 µg suppressed IFN-γ cytokine response compared with HBsAg-MF59 (*P*=0.1309 and *P*=0.0005, respectively) and HBsAg-Alum (*P*=0.0001) groups

Results from IFN-γ evaluation showed that all groups receiving a vaccine, including HBsAg-MF59 and HBsAg-Alum, demonstrated a significant increase, as compared with those receiving MF59, MF59+PPD1 µg, MF59+ PPD10 µg, and Alum, as well as PBS control groups (*P*<0.0005). Assessment of cytokine IFN-γ results in the HBsAg-Alum formulation group showed no significant difference as compared with the HBsAg-MF59 group (P=0.2300). Immunization with PPD formulated HBsAg-MF59 vaccine at 1 and 10 µg doses, suppressed IFN-γ compared with HBsAg-MF59 (*P*=0.1309 and *P*=0.0005, respectively) and HBsAg-Alum (*P*=0.0001) groups (Figure 1). 

***Results of IL-4 cytokine***

**Figure 2:**
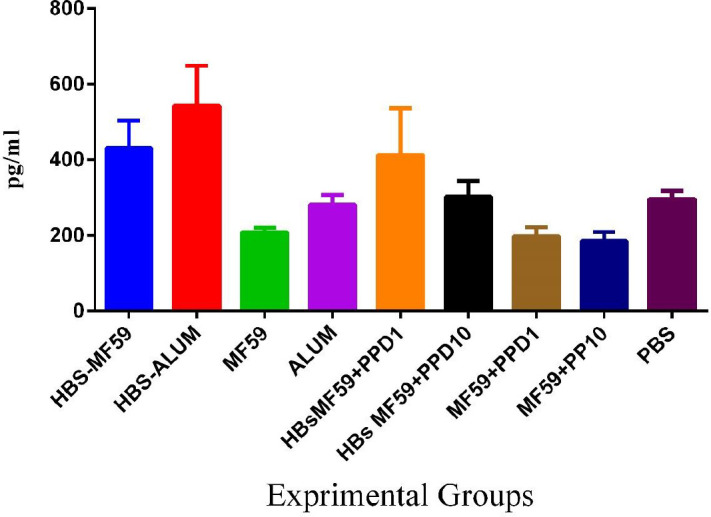
IL-4 cytokine response in the vaccinated mice. Assessment of cytokine IL-4 in the HBsAg-Alum group showed an increase as compared with HBsAg-MF59 formulation but at borderline (*P*=0.0553). Mice immunized with 10 µg PPD-formulated HBsAg-MF59 vaccine suppressed IL-4 cytokine response compared with HBsAg-MF59 (*P*=0.0378). Furthermore, mice immunized with the HBsAg-MF59 vaccine formulated in PPD at the doses of 1 and 10 µg suppressed IL-4 cytokine response compared with HBsAg-Alum (*P*=0.0158 and *P*=0.0001, respectively)

Mice immunized with HBsAg-MF59 and HBsAg-Alum showed an increase in IL-4, as compared with those immunized with MF59, MF59+PPD1 µg, MF59+PPD10 µg, and Alum, as well as PBS control groups ((*P*<0.0204). However, HBsAg-MF59 versus the PBS group was at borderline (*P*=0.0513). Assessment of IL-4 in HBsAg-Alum showed an increase as compared with HBsAg-MF59 formulation but at a borderline (*P*=0.0553). Mice immunized with the HBsAg-MF59 vaccine formulated in PPD at the dose of 10 µg suppressed IL-4 cytokine response in comparison with HBsAg-MF59 (*P*=0.0378). Furthermore, mice immunized with the PPD-formulated HBsAg-MF59 vaccine at the doses of 1 and 10 µg suppressed IL-4 cytokine response compared with the HBsAg-Alum group (*P*=0.0158 and *P*=0.0001, respectively) (Figure 2).

***Result of the IL-2/IL-4 cytokine ratio***

**Figure 3:**
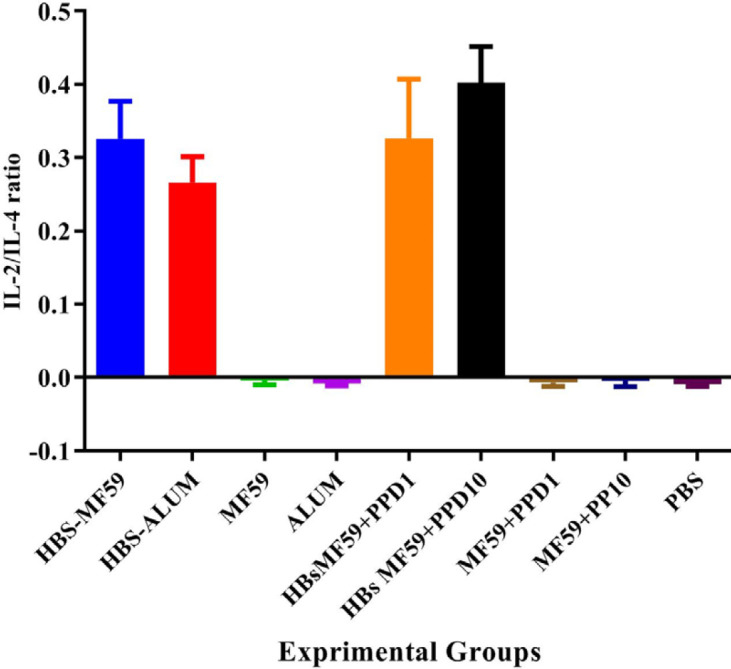
Results of IL-2/IL-4 cytokine ratio. Results from the IL-2/IL-4 ratio in HBsAg-MF59, HBsAg-Alum, HBsAg-MF59+ PPD1 µg, and HBsAg-MF59+PPD10 µg groups showed a significant increase compared with the control groups (*P*<0.0001). In addition, mice immunized with HBsAg-MF59+PPD10 µg showed a significant increase compared with those immunized with HBsAg-MF59 and HBsAg-Alum (*P*=0.0339 and *P*=0.0001, respectively). Furthermore, the HBsAg-MF59+PPD10 µg group showed a significant increase versus the HBsAg-MF59+PPD1 µg group (*P*=0.0374) (Figure 2)

Results from the IL-2/IL-4 ratio in HBsAg-MF59, HBsAg-Alum, HBsAg-MF59+ PPD1 µg, and HBsAg-MF59+PPD10 µg groups showed a significant increase, as compared with the control groups (*P*<0.0001). In addition, mice immunized with HBsAg-MF59+PPD10 µg showed a significant increase as compared with those immunized with HBsAg-MF59 and HBsAg-Alum group (*P*=0.0339 and *P*=0.0001, respectively). Furthermore, the HBsAg-MF59+PPD10 µg group showed a significant increase, compared with the HBsAg-MF59+PPD1 µg group (*P*=0.0374) (Figure 3). 

***Specific total IgG response***

**Figure 4:**
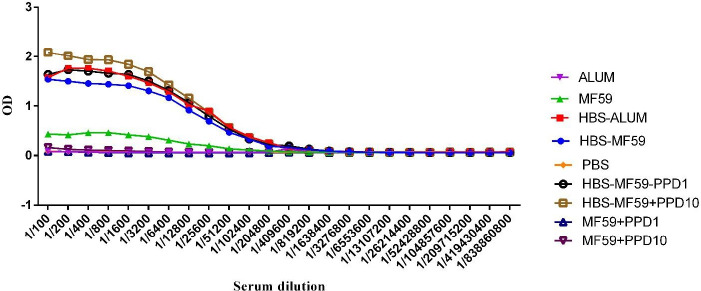
Results of specific total IgG of vaccinated mice two weeks after the final shot. Mice immunized with HBsAg-Alum two weeks after the final immunization showed a significant increase in IgG response versus the HBsAg-MF59 group at dilutions of 1/200 up to 1/800 (*P*<0.0041). Mice immunized with HBsAg-MF59 formulated in PPD 1 µg vaccine showed a significant increase versus the HBsAg-MF59 group at dilutions of 1/200 and 1/400 (*P*=0.0253). Mice immunized with HBsAg-MF59 formulated in PPD 10 µg vaccine showed a significant increase versus the HBsAg-MF59 group at dilutions of 1/100 up to 1/6400 (*P*=0.0281). Mice immunized with HBsAg-MF59 formulated in PPD 10 µg vaccine showed a significant increase versus the HBsAg-Alum group at dilutions of 1/100, 1/200, and 1/1600 (*P*=0.0332)

Mice immunized with HBsAg-MF59 and HBsAg-MF59 formulated in PPD 1 and 10 µg vaccines two weeks after final immunization showed a significant increase compared with control groups at dilutions of 1/100 up to 1/51200 (*P*<0.0395), while HBsAg-Alum group significantly increased IgG at dilutions of 1/100 up to 1/102400 compared with Alum and PBS control groups (*P*<0.0063). Mice immunized with HBsAg-Alum showed a significant IgG increase versus the HBsAg-MF59 group at dilutions of 1/200 up to 1/800 (*P*<0.0041). Mice immunized with PPD formulated HBsAg-MF59 1 µg group significantly showed an increase in IgG at dilutions of 1/200 up to 1/400 compared with the HBsAg-MF59 group (*P*=0.0253). On the other hand, immunized mice with 10 µg PPD-formulated HBsAg-MF59 showed a significant increase at dilutions of 1/100 up to 1/6400 compared with HBsAg-MF59 (*P*=0.0281) and HBsAg-Alum at dilutions of 1/100, 1/200, and 1/1600 (*P*=0.0332), respectively (Figure 4). 

***Results of long-lived specific IgG antibody responses on day 90 post-final immunization***

**Figure 5:**
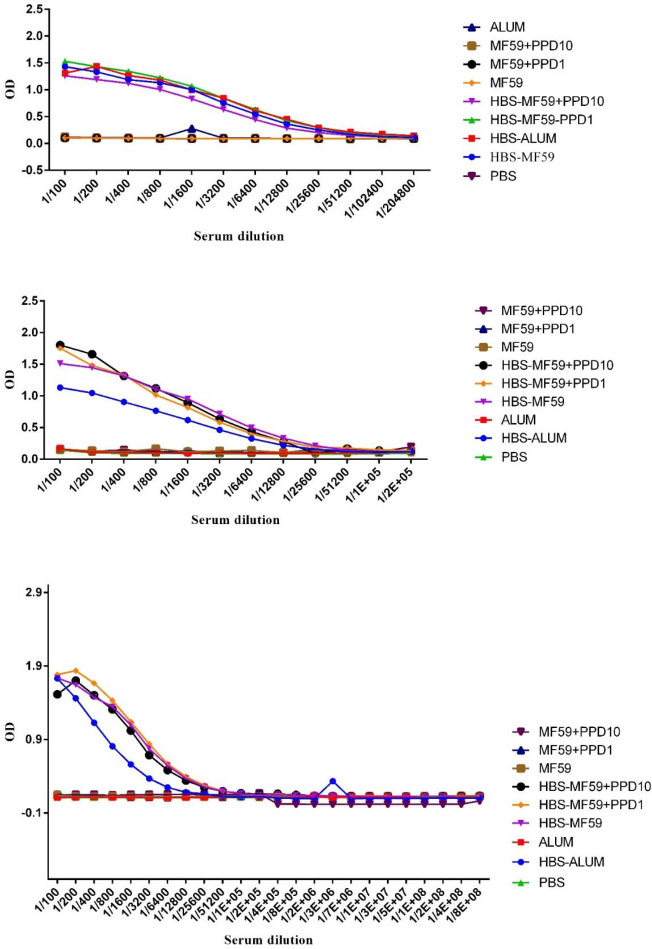
Results of long-lived specific total IgG antibody responses post final immunization. (A), Results of specific IgG response after 90 days post-final immunization. Mice immunized with HBsAg-Alum showed no significant difference in IgG response versus the HBsAg-MF59 group in any experimental dilutions (*P*>0.9118). Mice immunized with HBsAg-MF59 formulated in PPD 1 and 10 µg vaccine showed no significant difference compared with HBsAg-MF59 and HBsAg-Alum groups at any experimental dilutions (*P*>0.1153). (B), Results on day 150 post-final immunization. Mice immunized with HBsAg-MF59 at dilutions of 1/100 up to 1/3200 significantly increased IgG response in comparison with the HBsAg-Alum group (*P*<0.0288). Mice immunized at dilutions of 1/100 up to 1/1600 with HBsAg-MF59 formulated in PPD 1 and 10 µg (at dilutions of 1/100 up to 1/3200) showed a significant increase in the IgG response compared with HBsAg-Alum group (*P*<0.0222 and *P*<0.0059, respectively). Mice immunized with 1 and 10 µg of PPD-formulated HBsAg-MF59 vaccine significantly increased while comparing the HBsAg-MF59 group at a dilution of 1/100 (*P*=0.0390 and *P*=0.0048, respectively). (C), Specific IgG antibody responses on day 220 post-final immunization. At dilutions of 1/400 up to 1/6400, the results demonstrated that IgG was significantly increased in mice immunized with HBsAg-MF59 compared with HBsAg-Alum (*P*<0.0006). Mice immunized with HBsAg-MF59 formulated in PPD 1mg vaccine showed an increase versus the HBsAg-MF59 group at dilutions of 1/200 and 1/400 (*P*<0.1102). Mice immunized with HBsAg-MF59 formulated in PPD 10 µg vaccine showed a significant decrease versus the HBsAg-MF59 group at a dilution of 1/100 (*P*=0.0291). Mice immunized with HBsAg-MF59 formulated in PPD 1 and 10 µg vaccines showed a significant increase versus the HBsAg-Alum group at dilutions of 1/200 up to 1/6400 (*P*<0.0205)

Mice immunized with HBsAg-MF59, 1 and 10 µg PPD formulated HBsAg-MF59 vaccines showed a significant increase at dilutions of 1/100 up to 1/6400 versus control groups (*P*<0.0402), while dilutions of 1/100 up to 1/12800 in HBsAg-Alum group induced a significant increase versus control groups, Alum and PBS (*P*<0.0082). Mice immunized with HBsAg-Alum showed no significant difference in the IgG response versus the HBsAg-MF59 group in all experimental dilutions (*P*>0.9118). Mice immunized with HBsAg-MF59 formulated in PPD 1 and 10 µg vaccine showed no significant difference versus HBsAg-MF59 and HBsAg-Alum groups at all experimental dilutions (*P*>0.1153) (Figure 5A for day 90). 

***Results of long-lived specific IgG antibody responses on day 150 post-final immunization***

Mice immunized with the HBsAg-MF59 vaccine at dilutions of 1/100 up to 1/6400 showed significant increase versus MF59 and PBS (*P*<0.0130). Mice immunized with HBsAg-MF59+ PPD 1 and 10 µg showed a significant increase at dilutions of 1/100 up to 1/3200 versus their cognate control groups (*P*<0.0014). At dilutions of 1/100 up to 1/3200, mice immunized with the HBsAg-Alum group showed a significant increase versus Alum and PBS control groups (*P*<0.0087). Mice immunized with HBsAg-MF59 at dilutions of 1/100 up to 1/3200 showed a significant increase in IgG response versus the HBsAg-Alum group (*P*<0.0288). PPD 1 formulated HBsAg-MF59 immunization (at dilutions of 1/100 up to 1/1600) and 10 µg (at dilutions of 1/100 up to 1/3200) showed a significant increase in IgG response versus HBsAg-Alum group (*P*<0.0222 and *P*<0.0059, respectively). Mice immunized with HBsAg-MF59 vaccine formulated in PPD 1 and 10 µg showed a significant increase versus the HBsAg-MF59 group at dilution of 1/100 (*P*=0.0390 and *P*=0.0048, respectively) (Figure 5B for day 150).

***Results of long-lived specific IgG antibody responses on day 220 post-final immunization***

Mice immunized with HBsAg-MF59 (at dilutions of 1/100 up to 1/6400), HBsAg-MF59 formulated in PPD 1 µg (at dilutions of 1/100 up to 1/12800), and PPD 10 µg (at dilutions of 1/100 up to 1/6400) vaccines significantly increased IgG antibody responses (*P*<0.0404), while the HBsAg-Alum group showed a significant increase at dilutions of 1/100 up to 1/1600 versus Alum and PBS control groups (*P*<0.0001). Mice immunized with HBsAg-MF59 at dilutions of 1/400 up to 1/6400 showed a significant increase in the IgG response versus HBsAg-Alum (*P*<0.0006). Mice immunized with HBsAg-MF59 formulated in PPD 1 µg at dilutions of 1/200 and 1/400 vaccine showed an increase versus the HBsAg-MF59 group (*P*<0.1102). 

Mice immunized at dilution of 1/100 with HBsAg-MF59 formulated in PPD 10 µg vaccine showed a significant decrease versus the HBsAg-MF59 group (*P*=0.0291). Immunization of mice at dilutions of 1/200 up to 1/6400 with HBsAg-MF59 formulated in PPD 1 and 10 µg vaccines was accompanied by a significant increase versus the HBsAg-Alum group (*P*<0.0205) (Figure 5C for day 220).

***Specific IgG1 isotype***

**Figure 6:**
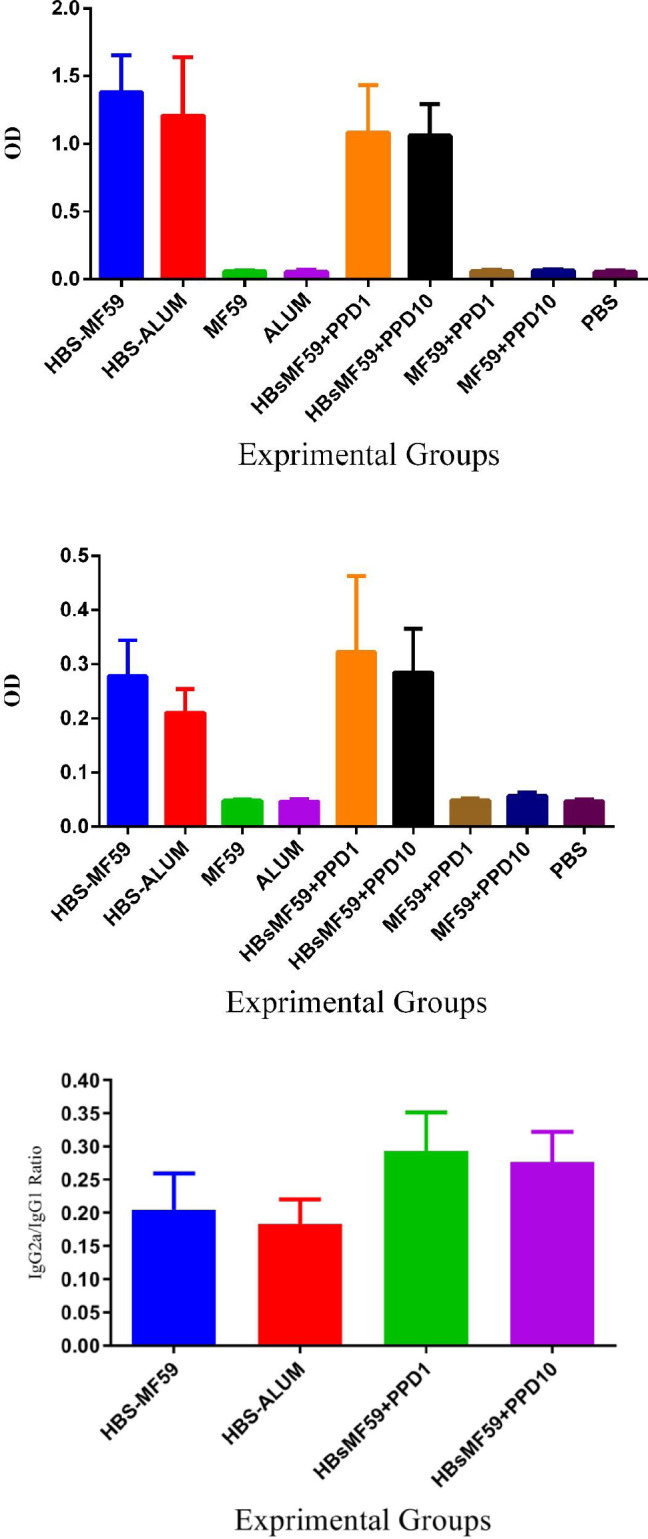
(A), The results of specific IgG1 isotype antibody after the third shot of experimental mice. Specific IgG1 isotype levels in HBsag-MF59 showed no significant difference versus HBsAg-Alum group (*P*=0.2317). Mice immunized with HBsAg-MF59 in PPD 10 mg showed a border line decrease in IgG1 level versus HBsAg-MF9 group (*P*=0.0648). In addition, HBsAg-MF59 in PPD-1 and 10mg vaccines showed no significant differences versus HBsAg-Alum group (*P*=0.7023). (B), Specific IgG2a isotype antibody after final immunization. Mice immunized with HBsAg-MF59 vaccine showed a significant increase versus HBsAg-Alum group (*P*=0.0190). In addition, mice immunized with HBsAg-MF59 formulated in PPD 10mg showed an increase versus HBsAg-MF59 group (*P*=0.0040). Mice immunized with HBsAg-MF59 formulated in PPD 1 and10mg showed an increase versus HBsAg-Alum group (*P*=0.0040). (C), The result of IgG2a/IgG1 ratio of experimental vaccinated mice. Results from the IgG2a/IgG1 ratio in HBsAg-MF59+PPD1µg and HBsAg-MF59+PPD10µg showed a significant increase versus HBsAg-Alum and HBsAg-MF59 groups (*P*<0.0345)

Results from specific IgG1 isotype antibody after the third shot demonstrated that mice immunized with HBsAg-MF59, HBsAg-Alum, and 1 and 10 µg PPD-formulated HBsAg-MF59 groups showed a significant increase compared with control groups (*P*<0.0001). Specific IgG1 isotype level in HBsAg-MF59 showed no significant difference versus HBsAg-Alum (*P*=0.2317). Mice immunized with HBsAg-MF59 in PPD 10 µg showed a borderline decrease in the IgG1 level versus the HBsAg-MF9 group (*P*=0.0648). In addition, HBsAg-MF59 in PPD-1 and 10 µg vaccines showed no significant differences versus the HBsAg-Alum group (*P*=0.7023) (Figure 6A, IgG1).

***Specific IgG2a isotype***

Results from the specific IgG2a isotype antibody demonstrated that mice immunized with HBsAg-MF59, HBsAg-Alum, and HBsAg-MF59 formulated in PPD 1 and 10 µg accompanied a significant increase as compared with the control groups (*P*<0.0136). Mice immunized with the HBsAg-MF59 vaccine showed a significant increase versus HBsAg-Alum (*P*=0.0190). In addition, mice immunized with HBsAg-MF59 formulated in PPD 10 µg showed an increase versus the HBsAg-MF59 group (*P*=0.0040). Mice immunized with HBsAg-MF59 formulated in PPD 1 and 10 µg showed an increase versus HBsAg-Alum (*P*=0.0040) (Figure 6B, IgG2a).

***IgG2a/IgG1 ratio***

Results from the IgG2a/IgG1 ratio in HBsAg-MF59+PPD1 µg showed a significant increase versus HBsAg-Alum and HBsAg-MF59 groups (*P*=0.0008 and *P*=0.0074, respectively). In addition, immunization with HBsAg-MF59+PPD10 µg showed a significant increase, as compared with the HBsAg-Alum and HBsAg-MF59 groups (*P*=0.0042 and *P*=0.0345, respectively). There was no significant difference between HBsAg-MF59+PPD1 µg and Figure 6C, IgG2a/IgG1 ratio.

## Discussion

The commercial Alum-based HBsAg vaccine is able to protect the body through induction of stimulating Th2 response and humoral immune responses. However, this vaccine is not able to trigger Th1 and cellular immune responses against HBsAg in human and animal models (16). 

In this study, recombinant HBsAg was formulated in MF59 adjuvant and then as a co-adjuvant, PPD was added to the vaccine formulation to boost the specific immune responses against the HBsAg vaccine. In addition, long-lived humoral immune responses were followed up until day 220 after the final vaccination. Results from the IFN-γ assay after immunization with the HBsAg-MF59 vaccine showed no positive effect versus the Alum-based vaccine. Surprisingly, using PPD in the formulation of the HBsAg-MF59 vaccine, a considerable suppression in IFN-γ cytokine release was observed versus HBsAg-MF59 and even the HBsAg-Alum group. However, mounting evidence has shown that PPD can induce Th1-related immune responses and also dendritic cell maturation and activation as the most important cells in the triggering and activation of T cells (15, 17, 18). Herein, PPD in the vaccine formulation did not have any significant effect on the IFN-γ cytokine level compared with the HBsAg vaccine and even suppressed the response. Our previous study on PPD in HBsAg-Alum, also, did not induce any significant effect on IFN-γ cytokine response (19). Various studies have shown the potency of PPD in the induction of cellular immune responses and IFN-γ cytokine release (20, 21). Herein, the suppression of IFN-γ cytokine response may be due to the antigenic competition between PPD and HBsAg which might have led to a suppressive effect, however, this needs to be further studied.

Results from the IL-4 cytokine evaluation showed a borderline suppression in the HBsAg-MF59 vaccine versus the HBsAg-Alum group. In addition, mice immunized with HBsAg-MF59 plus PPD10 µg in the vaccine formulation showed a significant suppression in IL-4 cytokine response, as a criterion of the Th2 pattern (22) versus the HBsAg-MF59 group. This finding suggests that PPD in the HBsAg vaccine formulation suppressed the Th2 pattern and inversely it may show improvement in Th1 response. In other studies, HBsAg-Alum formulation with PPD showed a suppressive effect on IL-4 cytokine response, as consistent with this study (19).

Results from the IL-2/IL-4 ratio showed a significant increase in the HBsAg-MF59-PPD vaccine versus the HBsAg-MF59 group and confirms Th1 polarization. This finding may be due to the different levels of suppression activity of PPD on each cytokine response, because the most and lowest suppression activities were detected in IFN-γ and IL-4, and in IL-2, respectively. Thus, PPD may improve Th1 response, not by increasing IFN-γ cytokine release, but through suppression of IL-4 cytokine response and increase in the IL-2/IL-4 ratio versus the HBsAg-MF59 vaccine.

Results from humoral immune responses following the third immunization with 10 µg PPD1 formulated HBsAg-MF59 vaccine was accompanied by a significant increase versus the HBsAg-MF59 which shows the potency of PPD in the HBsAg vaccine formulation in the context of improving humoral immune responses. In another finding, HBsAg-Alum showed a significant increase in the IgG response versus the HBsAg-MF59 group, but a significant increase was observed versus the HBsAg-Alum group when PPD at the dose of 10 µg was added to the HBsAg-MF59 vaccine formulation. This finding shows that the Alum-based vaccine has a higher potency compared with HBsAg-MF59 in the induction of humoral response, 4 weeks after the final boosting. Yet, HBsAg-MF-59 plus PPD 10 µg is more potent than the HBsAg-Alum vaccine in the induction of humoral immune responses. In the other words, PPD induces a positive effect in the induction of humoral immune responses in the HBsAg-MF-59 vaccine. 

A study on *Pasteurella multocida* serotype A:1 and A:4 whole cell antigens which were formulated by PPD and Montanide ISA-206 adjuvants showed a positive effect of PPD on the humoral immune responses in a chicken model which was in agreement with the result obtained in the present study (23).

Results from 90 days after the final boosting suggested a comparable humoral immune response in all HBsAg vaccinated groups. However, at day 150 of the final boosting, the HBsAg-MF59 vaccine induced more potent humoral immune responses than HBsAg-Alum, and PPD at the doses of 1 and 10 µg increased the potency of HBsAg-MF59 inducing specific IgG responses compared with the HBsAg-MF59 vaccine. In addition, at day 220 of the final immunization, the HBsAg-MF59 vaccine significantly increased the specific IgG responses versus the HBsAg-Alum group. Furthermore, PPD at the doses of 1 and 10 µg increased the potency of the HBsAg-MF59 vaccine inducing specific IgG responses compared with the HBsAg-MF59 vaccine. These findings first showed that the MF59-based vaccine has more potency than the HBsAg-Alum vaccine inducing long-lived specific IgG responses, and secondly PPD in the HBsAg-MF59 vaccine formulation increased the vaccine potency in the induction of long-lived humoral response. In a study carried out by Zadon *et al*., the formulation of *Pasteurella Multocida* in PPD and Montanide ISA 206 as an oil-based adjuvant showed an improvement in the humoral immune response up to 42 days post the last vaccination of chickens (23). This finding showed the potency of PPD in the induction of long-term antibody responses as shown in the HBsAg vaccine model.

In a study, immunization with MF59- and Alum-adjuvanted hepatitis B containing surface and pre-S2 antigens showed that the MF59-adjuvanted vaccine had higher potency than the Alum-based vaccine inducing specific IgG responses (24). Research in adults with randomized control on Alum and /or MF59 adjuvant containing hepatitis B vaccine showed that the vaccine formulated in MF59 adjuvant was accompanied by higher potency than Alum-based vaccine inducing specific IgG response (25). A study on MF59-formulated HBsAg vaccine on male baboons showed that the vaccine had more potency than the Alum-based vaccine inducing specific IgG responses, and monitoring the primates seven months after the final shot showed that HBV/MF59 elicited a higher titer than the vaccine with Alum basis (26). The results from this study on IgG response are in agreement with our findings for short-term and long-term monitoring of IgG responses. Here for the first time, we showed that PPD in the HBsAg-MF59 vaccine formulation is able to increase humoral immune responses for short- and long-term post-final vaccination. Specific IgG1 and IgG2a isotypes analysis showed that PPD in the HBsAg-MF59 vaccine decreased IgG1 response and increased IgG2a response versus the HBsAgMF59 vaccine. In addition, the HBsAg-MF59 vaccine significantly increased the IgG2a response compared with HBsAg-Alum. Furthermore, results from the IgG2a/IgG1 ratio of HBsAg-MF59 formulated in PPD1 µg and PPD10 µg showed a significant increase versus the HBsAg-MF59 group. Since IgG2a is a criterion of Th1 activity (2), this finding showed that the MF59-adjuvanted vaccine has more potency than the Alum-based vaccine inducing Th1 response, and PPD in the vaccine formulation may improve this effect. Considering the suppression effect of PPD on IL-4 and IFN-γ cytokines response, it seems that other immunologic mechanisms, such as T follicular helper cell (Tfh), are involved in the improvement of the humoral immune response. Various studies demonstrated that mycobacterium antigens can induce IL-21 cytokine release as the most important cytokine of Tfh and thereby directly controls the germinal center B cell function and plasma cell formation and finally improvement of humoral immune responses (27-30). 

## Conclusion

Altogether, our results revealed that the HBsAg-MF59 vaccine was superior to HBsAg-Alum inducing long-lived humoral immune responses. In addition, PPD in the HBsAg vaccine formulation suppressed IL-4 and IFN-γ responses but increased the IL-2/IL-4 ratio, IgG2a, and IgG2a/IgG1 responses which may show a positive effect on the Th1 polarization. Furthermore, PPD leads to a more potent long-lived IgG response in the HBsAg vaccine. It seems that PPD may be useful as a component of a complex adjuvant.

## Authors’ Contributions

MEM Conceived and designed the study; RM, FN, MIM, BG, NR, FA, MAS, and MEM Performed animal experiments, immunoassay, data analysis, and draft manuscript preparation; FN, AK, and MEM Critically revised the paper; RM, FN, MIM, BG, AK, NR, FA, MAS, and MEM Supervised the research; RM, FN, MIM, BG, AK, NR, FA, MAS, and MEM Approved the final version to be published.

## Conflicts of Interest

The authors declare no conflicts of interest.
